# Protocol of an expertise based randomized trial comparing surgical Venae Sectio versus radiological Puncture of Vena Subclavia for insertion of Totally Implantable Access Port in oncological patients

**DOI:** 10.1186/1745-6215-9-60

**Published:** 2008-10-24

**Authors:** Philip Knebel, Lars Fischer, Eva Cremonese, Ruben Lopez-Benitez, Ulrike Stampfl, Boris Radeleff, Hans-Ulrich Kauczor, Markus W Büchler, Christoph M Seiler

**Affiliations:** 1 Department of General, Visceral and Transplantation Surgery, University of Heidelberg, Germany; 2 Department of Diagnostic Radiology, University of Heidelberg, Germany

## Abstract

**Background:**

Totally Implantable Access Ports (TIAP) are being extensively used world-wide and can be expected to gain further importance with the introduction of new neoadjuvant and adjuvant treatments in oncology. Two different techniques for the implantation can be selected: A direct puncture of a central vein and the utilization of a Seldinger device or the surgical Venae sectio. It is still unclear which technique has the optimal benefit/risk ratio for the patient.

**Design:**

A single-center, expertise based randomized, controlled superiority trial to compare two different TIAP implantation techniques. 100 patients will be included and randomized pre-operatively. All patients aged 18 years or older scheduled for primary elective implantation of a TIAP under local anesthesia who signed the informed consent will be included. The primary endpoint is the primary success rate of the randomized technique. Control Intervention: Venae Sectio will be employed to insert a TIAP by a surgeon; Experimental intervention: Punction of V. Subclavia will be used to place a TIAP by a radiologist. Duration of study: Approximately 10 months, follow up time: 90 days.

**Organisation/Responsibility:**

The PORTAS 2 – Trial will be conducted in accordance with the protocol and in compliance with the moral, ethical, and scientific principles governing clinical research as set out in the Declaration of Helsinki (1989) and Good Clinical Practice (GCP). The Center of Clinical Trials at the Department of Surgery, University Hospital Heidelberg is responsible for design and conduct of the trial including randomization and documentation of patients' data. Data management and statistical analysis will be performed by the independent Institute for Medical Biometry and Informatics (IMBI), University of Heidelberg.

**Trial Registration:**

The trial is registered at ClinicalTrials.gov (NCT00600444).

## Background

The first implantation of a TIAP was performed and described by Niederhuber et al. in 1982. Since then the insertion of a TIAP is a routinely employed technique in patients who need a safe and permanent venous access for chemotherapy, parenteral nutrition, recurrent blood sampling or other reasons [1]. This system needs no external dressing, allows the patient normal physical activity, is probably less prone to infectious complications and will minimize the occlusion rate of the catheter compared to non-totally implantable catheters [1]. TIAPs are being extensively used world-wide and an increase in the number of port placements can be expected. In Germany 70233 TIAP inpatient implantations were performed in 2006 (Federal Statistical Office, Wiesbaden, Germany). The number of TIAP implantations increased constantly at our Department from 169 in 1997 to 754 in 2007.

Today two different approaches to implant a TIAP are usually employed. Venae Sectio (VS) of the cephalic vein performed predominantly by surgeons and Puncture of Vena Subclavia (PVS) performed by interventional radiologists or surgeons. While most common complications can be observed with both techniques such as "pinch off" phenomena, kinking or dislocation of the catheter, subcutaneous hematoma, nerve palsy and wound infection, there are specific risks only associated with PVS like pneumo- and haematothorax [1,2].

Correct placement of the TIAP in the superior Vena Cava is mandatory for optimal and safe function of the central venous access. The median success rate of TIAP implantation via the conventional approach by transsection of the cephalic vein is 80% in various prospective and retrospective trials [2]. In contrast PVS achieved a success rate between 98 to 100%, up to now in retrospective studies [3-8].

## Study design

### Aim of study

The comparison of VS performed by a surgeon versus PVS performed by an interventional radiologist.

### Number of patients needed

**Figure 1:**
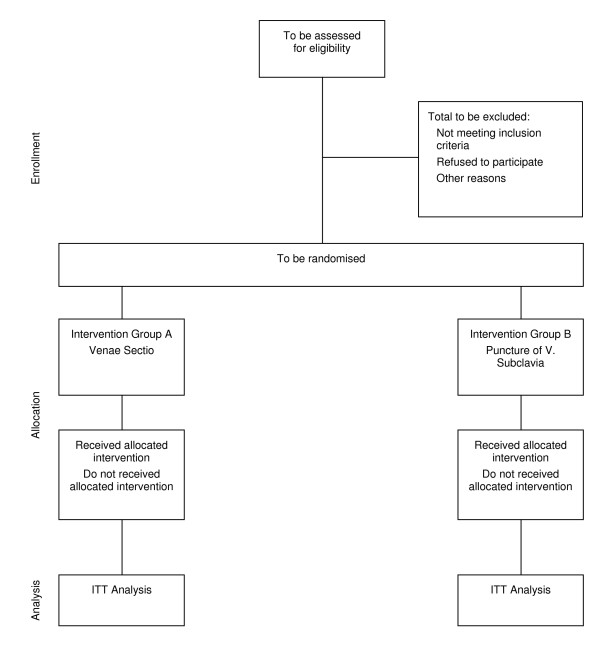
Flowchart according to CONSORT.

A review of published literature showed a median primary success rate of 80% for the VS in retro- and prospective studies [1,2,9-15]. PVS achieved a median primary success rate of 99% in various retrospective studies [3-8]. The group size for double sided testing was calculated with Simple Interactive Statistical Analysis (SISA) [16]. If the difference in success rate is 19% (80% vs. 99%) there will be an 80% chance that a trial involving 100 patients (50 per group) could detect a significant difference at an alpha level of 5% (Figure 1). We decided to calculate the sample size on the results of previous studies instead of calculating the sample size on a defined minimal clinical important difference because all mentioned previous studies showed a consistent median difference of 19%. This offers the opportunity to test these results the first time in a RCT setting with an economical sample size.

### Eligibility

#### Inclusion criteria

• Benign and malignant diseases which demand a safe and permanent venous access, e.g. for chemotherapy or parenteral nutrition

• Age 18 years or older

• Patients scheduled for primary elective implantation of TIAP under local anesthesia

• Informed consent

#### Exclusion criteria

• Participation in another intervention-trial with interference of intervention and outcome of this study

• Lack of compliance (assessed by the trial investigator)

• Impaired mental state or language problems (Patient is not able to read German)

• Patients with known allergy to contrast agent

#### Subject withdrawal criteria

• At their own request or at request of the legal representative

• If, in the investigator's opinion, continuation of the trial would be detrimental to the subject's well-being

All withdrawn patients will be reported in the final results to guarantee maximum transparency.

### Consent

Patients who are assigned for port-catheter-system implantation either at the Department of Radiology or Surgery, University hospital of Heidelberg, will be screened for their eligibility and informed about the PORTAS 2 trial during a visit prior to treatment. The study procedure, risks, benefits and data management will be clarified in detail before the patients are asked to give their informed consent. After inclusion of the patient his personal data (height (cm), weight (kg), gender, smoking customs, Karnowsky-Index (0 – 100%), medication of immunsuppresion, antibiotics (yes/no), chemotherapy (yes/no)) will be recorded into the CRF (Table 1).

**Table 1:** Study visit schedule

			Follow-up
			
	Day of screening	Day of operation	Visit 1 (day 90 post OP) by phone
Past medical history*	X		

Informed consent	X		

Personal data**	X		

Examination of primary endpoints:			
• Success of randomized implantation technique		X	

Examination of secondary endpoints:			
• Peri- and postoperative complications		X	X
• Times of port implantation procedure		X	
• Dose of radiation		X	

Savety criteria AE, SAE (2.6)		X	X

* study-relevant past medical history, past surgical history

** height (cm), weight (kg), gender, smoking customs, Karnowsky-Index, medication of immunsuppresion, antibiotics, chemotherapy

### Randomization and procedures for minimizing bias

#### Minimizing bias

To achieve comparable groups for known and unknown risk factors randomization will be performed as unstratified block randomization with random block sizes in a 1:1 allocation ratio. Allocation to treatment group will be carried out by the randomization software RITA^®^[17]. 110 patients will be recruited according to the sample size calculation. Randomization will be performed by a study nurse of the Clinical Study Center Surgery (KSC) expertise based to the Department of Surgery for patients in group A and to the Department of Radiology for patients in group B. Randomization will be carried out after patient signed the informed consent. Intervention will be scheduled 1–4 days after inclusion depending on the earliest operation appointment possible.

#### Minimizing treatment bias

All physicians who participate in this trial will be trained and updated every 3 months to guarantee comparable treatment of patients. Special manuals will be used in the operation and the radiology room to reduce error. The same TIAP device will be implanted in all patients (INTRAPORT II Keramic^® ^by Fresenius Kabi). Antibiotic prophylaxis will only be given to patients with risk for endocarditis according to the local standards or to patients scheduled for a chemotherapy < 5 days after implantation.

#### Minimizing measurement bias

A study nurse will document and monitor the procedure in the operating theatre or radiological intervention room. Blinding is not possible due to the nature of surgery and the allocation to different departments.

### Study treatment

All patients will be positioned on the table in a five degree reverse Trendelenburg's position. The neck, chest and shoulders of the patients will be prepared and draped in the customary sterile manner.

The left V. Subclavia/V. Cephalica will be preferred except for one of the following cases:

• patient suffers from breast cancer on the left side

• patient is left-handed

• prior evidence that left V. subclavia is closed by a thrombosis

• patient's wish

• patient had port catheter on left side before

According to the allocation the procedure will be continued:

#### Intervention-group A (Venae Sectio performed by a surgeon)

**Figure 2:**
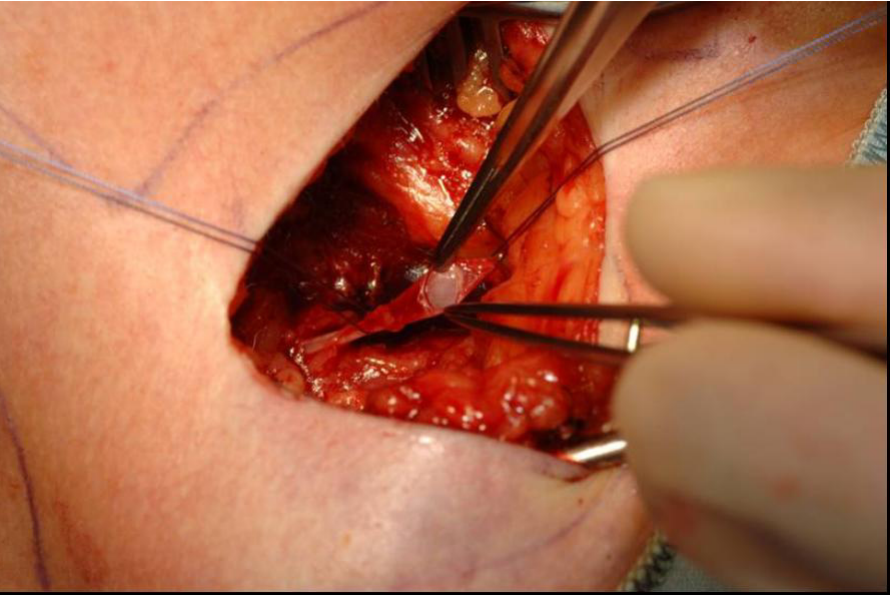
Venae Sectio – incision of cephalic vein.

**Figure 3:**
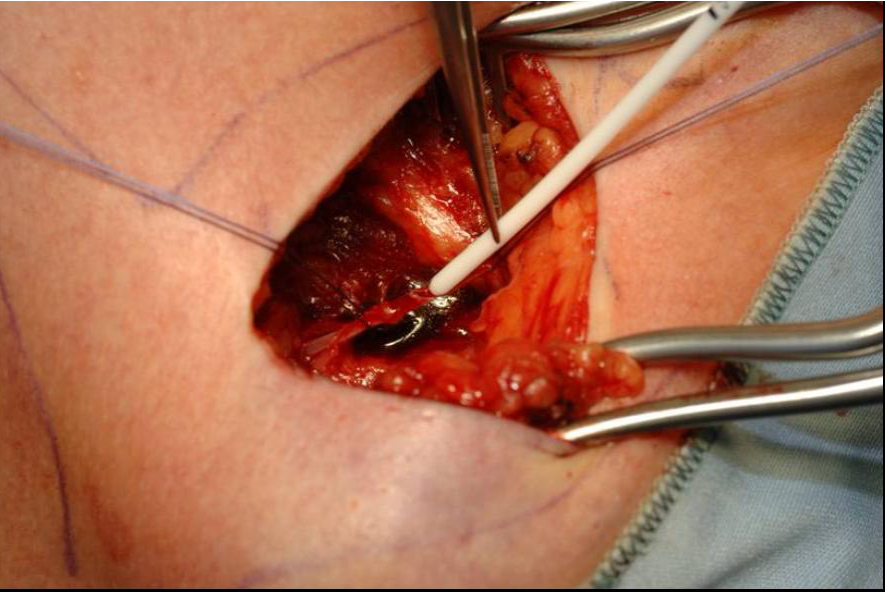
Venae Sectio – introducing of TIAP catheter.

Local anesthesia will be infiltrated in sterile fashion into skin and subcutaneous layer and skin incision will be performed 4 cm inferolaterally over the deltoideo-pectoral sulcus region. The cephalic vein is to be exposed. The cephalic vein will be ligated distally and encircled cranially with a reabsorbable suture. The vein will be cross-sected ventrally and the catheter flushed with heparinized saline, then introduced (figure 2, 3). The introduction of the catheter may be supported by use of a guiding wire, vein dilatator and peel away sheath if necessary. After placement of the catheter the correct positioning will be controlled via fluoroscopy (tip of catheter in the V. cava superior just to the tune of the bronchial bifurcation). The catheter will be connected to the port chamber. Using the same incision, a subcutaneous pocket will be prepared on the pectoral fascia. The port chamber will be fixed on the fascia of the pectoral muscle with three single non absorbable sutures. The wound will be closed with an absorbable subcutaneous suture and skin will be closed by a non absorbable intracutaneous suture. Unhindered flow for blood withdrawal and infusion is verified by cutaneous puncture (Huber needle). To complete the procedure the system is blocked with 2–4 ml heparinized saline (100 I.E./ml).

#### Intervention-group B (Punction of V. Subclavia by a radiologist)

**Figure 4:**
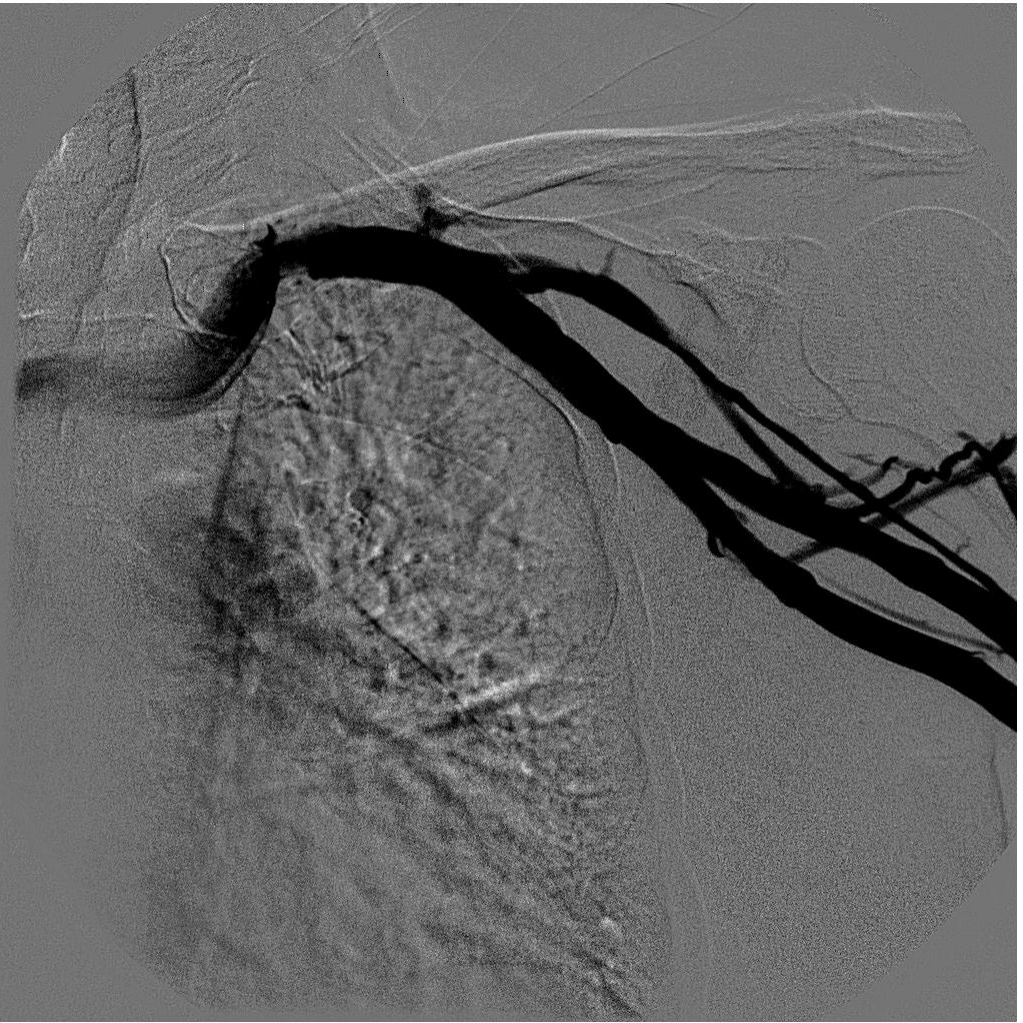
Puncture of V. Subclavia – demonstration of V. Subclavia with contrast agent.

**Figure 5:**
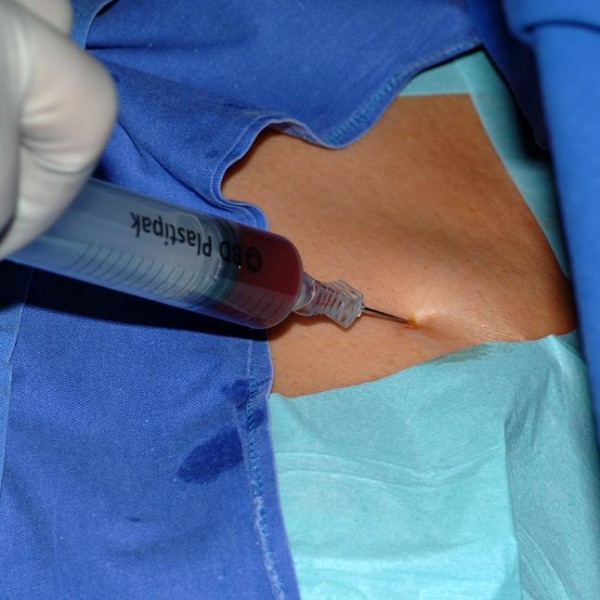
Puncture of V. Subclavia – puncture.

**Figure 6:**
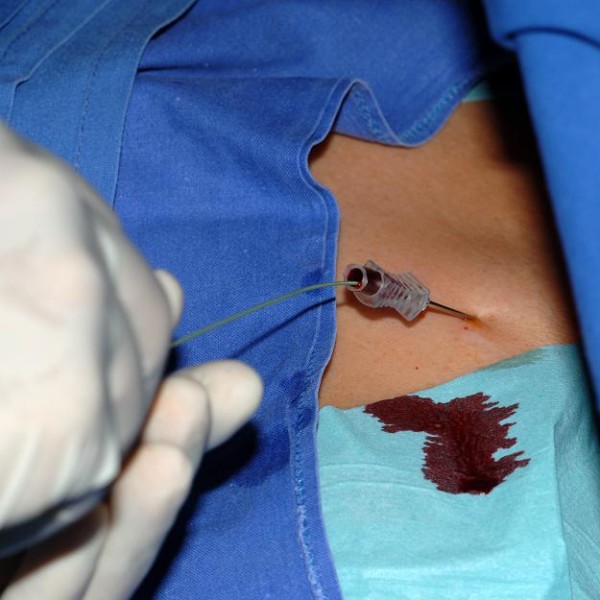
Puncture of V. Subclavia – introducing guiding wire (Seldinger technique).

**Figure 7:**
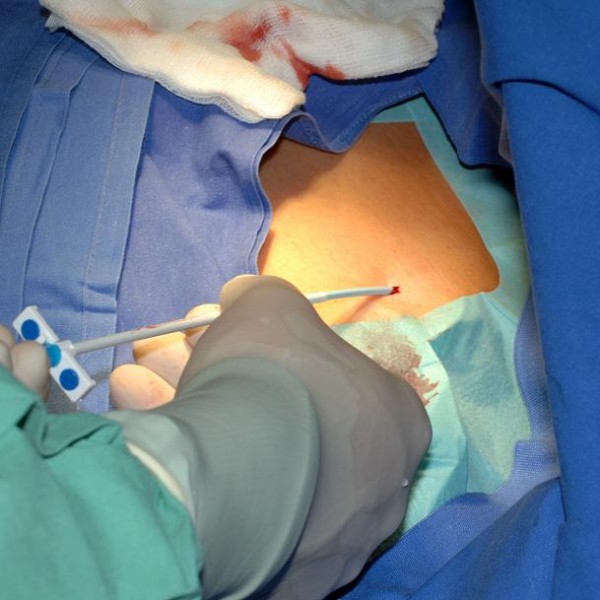
Puncture of V. Subclavia – introducing peel away sheath.

Patients receive a peripheral venous catheter on the side of planned puncture location for the administration of contrast agent (Ultravist^® ^370 by Bayer) and the verification that the V. subclavia is not closed by thrombosis (road-map technique, figure 4). Local anesthesia will be introduced in sterile fashion into skin and periost of the clavicula. Location of the puncture will be marked between proximal and medial third of the clavicula (figure 5). A ca. 2 cm wide skin incision will be performed above puncture location. In the following the V. Subclavia is punctured under fluorscopy using the Seldinger technique and a guiding wire is introduced (figure 6, 7). An introducer sheath is passed via the guiding wire into the V. subclavia. The guiding wire is removed and the port catheter may be introduced through the introducer sheath. Correct position is verified by fluoroscopy. A second ca. 3,5 cm wide skin incision is performed 1 cm below the first incision parallel to the clavicula. A subcutaneous pocket will be prepared on the pectoral fascia. The port chamber will be fixed on the fascia of the pectoral muscle with three single non absorbable sutures. The wound will be closed with an absorbable subcutaneous suture and skin will be closed by a non absorbable intracutaneous suture. Correct position of port catheter is checked again by fluorscopy (Tip of catheter in the V. cava superior just to the tune of the bronchial bifurcation). Flow for blood withdrawal and infusion is tested via cutaneous puncture (Huber needle). To complete the procedure the system is blocked with 2–4 ml heparinized saline (100 I.E./ml).

### Primary and secondary endpoints

#### Primary endpoint

The primary endpoint will be the success rate of the randomized implantation technique.

#### Definition of the primary endpoint

Primary success is defined as the correct position of the catheter in the V. Cephalica/V. Subclavia on the intended side controlled intraoperatively by radiography and correct function verified by drawing blood and infusion of fluid.

#### Assessment of the primary endpoint

The primary success will be assessed postoperatively by the responsible physician in the case report file (CRF) and will be confirmed by an independent study nurse and will be compared to the operation report. A copy of the intraoperative radiography showing the right position of the catheter will be saved in the digital radiological picture viewer software Centricity^®- ^used routinely by the University Hospital of Heidelberg.

#### Secondary endpoints

##### Perioperative complications

• pneumothorax

• hematothorax

• intraoperative lesion of nerves

• dislocation of the catheter or the port chamber

• intolerance of contrast agent

##### Postoperative complications

• thrombosis

• postoperative bleeding

• hematoma

• disconnection or breakage of the catheter

• extravasation of injected fluid

• wound infection

• cutaneous necrosis

Definitions are shown in Table 2 and Table 3

**Table 2:** Definitions of perioperative complications.

Pneumothorax	Radiological findings
Hematothorax	Radiological findings or sonografic findings

Intraoperative lesion of nerves	Clinical diagnosis or EMG findings

Dislocation of the catheter or the port chamber	Radiological finding

Intolerance of contrast agent	Any allergic reaction of contrast agent which requires any application of drugs

**Table 3:** Definitions of postoperative complications.

Thrombosis	Sonographic findings or phlebography
Postoperative bleeding	Clinical diagnosis during reoperation

Hematoma	Clinical diagnosis, no reoperation necessary

Disconnection or breakage of the catheter	Radiological findigs, findings after explantation

Extravasation of injected fluid	Radiological findings or clinical diagnosis

Wound infection	Clinical diagnosis. Reopening of wound necessary or antibiotic treatment.

Catheter sepsis	Two or more of the following symptoms:
	• temperature over 38.3°C or under 36°C
	• heart frequency over 90 beats per minute
	• breath frequency over 20 breaths per minut, PaCO2 < 32 mmHg (spontan breathing) or PaO2/FiO2 < 200 mmHg (mechanical ventilation)
	• Total peripheral WBC count > 12 G/L or WBC < 4.0 G/L or > 10% immature neutrophils (bands), regardless of total peripheral WBC count
	• Plasma C-reactive protein > 2 SD above normal value
	**AND**
	Positive findings in bacteriology of the Port catheter pike

Cutaneous necrosis	Clinical diagnosis or histological finding

##### Assessment

1. Perioperative complications of port implantation will be recorded with tick boxes at day of operation by an independent study nurse.

2. Postoperative complications of port implantation will be recorded with tick boxes at day of operation and visit 1 (90 days after operation) after a standardized telephone interview by a study nurse. A confirmation by the responsible family physician will be requested by any abnormality reported by the patient.

The duration of port implantation procedure

• Time from first skin incision to last knot of intracutaneous suture

• Time from patient entering until patient leaving intervention room

##### Assessment

Both times are recorded by an independent study nurse in the CRF at the day of operation.

##### Dose of radiation

Definition: Product of dose rate and surface of radiation (Gy × cm^2^).

##### Assessment

The value will be copied from the display of the used radioscopy device by a study nurse and recorded in the CRF.

### Safety aspects

#### Specification of safety variables

##### Training for surgeons

For surgeons/radiologists and tutors (senior surgeons/radiologists) who operated 25 or fewer ports so far the exact number of operated ports will be noted in the CRF. Surgeons and tutors (senior surgeons) who have performed more than 25 port operations so far will be classified in one of the following categories: 26–30; 31–35; 36–40; 41–45; 46–50; > 50 operated ports and recorded in the CRF.

##### Concomitant medication

Concomitant medication will not be recorded because the primary success rate of the two implantation techniques is a local and technical endpoint. Therefore, a systemic pharmacological interaction with the medication of the patient will be very unlikely.

##### Past medical history

Prior and concomitant illness of the patients will be documented in the CRF. The category of the primary disease (reason for port-catheter implantation) is one of the variables to be analyzed for baseline comparability.

##### Adverse events and serious adverse events

AEs will be reported to the principal investigator in regular intervals during the course of the study. Symptoms anticipated by chemotherapy and progression of malignant illness will not be recorded as AEs as they are not likely related to the surgical implantation technique.

SAEs which meet one of definitions of the secondary endpoints are treated as SAEs regarding their documentation but do not have to be reported to the sponsor (University Hospital of Heidelberg) and principal investigator (Prof. Dr. MW Büchler, Chairman of the Department of General, Visceral and Transplantation Surgery, University Hospital of Heidelberg) within 24 h. They will be reported to the principal investigator in regular intervals throughout the study. The surgical and radiological trial coordinator will also cross check the SAEs/AEs of all patients.

### Analysis

Comparisons will be made of the primary endpoints of both intervention groups for all randomized patients who underwent surgery for TIAP implantation. Patients will be analyzed as randomized. This is in line with the intention-to-treat principle [18]. In addition, a per-protocol analysis will be performed.

The outcome measures of the primary endpoint will be tested for significance with the chi-square test with continuity correction. Fisher's exact test will be used instead if one or more expected cell counts are less than five. No stratification will be used. The estimated odds ratio of primary success will be presented together with a 95% confidence interval. A secondary analysis will be performed using a multiple regression model including treatment group, age, body mass index, surgeon's experience and Karnofsky Index as predictors. All predictors except for treatment group will be used as continuous variables.

Patients with missing information regarding primary success will be considered as failures in all analyses of primary success except for one sensitivity analysis in which these patients will be excluded.

All statistical analyses will be performed using SAS^® ^software, Version 9.1 (or higher) of the SAS System for Unix (SAS Institute Inc., Cary, NC, USA).

### Study organization

All patients scheduled for a primary TIAP system implantation procedure in the Outpatient-Clinic of the Department of Surgery or Radiology, University Hospital of Heidelberg, will be referred to and screened by members of the Clinical Study Center Surgery (KSC). The result of the screening will be recorded in the screening-log.

Approximately 700 patients per year undergo a TIAP system implantation at the Outpatient-Clinic of the Department of Surgery and Radiology at the University of Heidelberg. The estimated time frame to randomize 110 patients will be approximately 6 months.

Sponsor of the PORTAS 2 trial is the University Hospital of Heidelberg.

The independent data management and statistical analysis will be carried out by the Institute of Medical Biometry and Informatics (IMBI) of the University of Heidelberg according to a prespecified Statistical Analysis Plan.

The principal investigator has the right to terminate the trial and to remove all trial material from the trial centre at any time in consultation with the Clinical Study Team Leader and the Biostatistician. Reasons that may require a termination of the trial include the following:

• The incidence or severity of adverse events in this trial indicates a potential health hazard caused by the study treatment

• It appears that patient's enrolment is unsatisfactory with respect to quality or quantity or data recording is severely inaccurate or incomplete

• External evidence that renders the necessity to terminate the trial

### Financial support

The trial will be sponsored in equal shares by a grant of Fresenius Kabi AG ^© ^and the regular research budget (State of Baden-Württenberg) of the Clinical Study Center Surgery (KSC), Department of General, Visceral and Transplantation Surgery of the University of Heidelberg.

## Competing interests

The Clinical Study Center received a grant from Fresenius Kabi AG ^©^. There are no restictions on the publications.

## Authors' contributions

PK, LF and CMS are responsible for the study design, definitions of the primary and secondary endpoints and preparation of the protocol. CMS is responsible for the sample size calculation. EC carried out the literature research. RL, US and BR are responsible for the radiological intervention arm and supported study planning. All authors read and approved the final manuscript.
